# Phytochemical Profiling, Antioxidant Capacity, and α-Amylase/α-Glucosidase Inhibitory Effects of 29 Faba Bean (*Vicia faba* L.) Varieties from China

**DOI:** 10.3390/biology14080982

**Published:** 2025-08-02

**Authors:** Ying Li, Zhihua Wang, Chengkai Mei, Wenqi Sun, Xingxing Yuan, Jing Wang, Wuyang Huang

**Affiliations:** 1 School of Ocean Food and Biological Engineering, Jiangsu Ocean University, Lianyungang 222005, China; hijoly@163.com (Y.L.); 2023220828@jou.edu.cn (Z.W.); 2 Institute of Agro-Product Processing, Jiangsu Academy of Agricultural Sciences, Nanjing 210014, China; 3 College of Chemical Engineering, Nanjing Forestry University, Nanjing 210037, China; 15519369907@njfu.edu.cn (C.M.); swenq@njfu.edu.cn (W.S.); 4 Institute of Industrial Crops, Jiangsu Academy of Agricultural Sciences, Nanjing 210014, China; yxx@jaas.ac.cn; 5 School of Food and Biological Engineering, Jiangsu University, Zhenjiang 212013, China

**Keywords:** faba beans, variety differences, geographical distribution, L-DOPA, phenolics, flavonoids

## Abstract

Faba bean (*Vicia faba* L.), a nutrient-rich legume beneficial to human health, is valued for its high L-3,4-dihydroxyphenylalanine (L-DOPA) and phenolic content. This study investigated phytochemical diversity and bioactivity across 29 Chinese faba bean varieties. Phenolics were profiled using ultrahigh-performance liquid chromatography with quadrupole time-of-flight mass spectrometry (UPLC-QTOF-MS) and quantified via high-performance liquid chromatography (HPLC). Antioxidant capacity was evaluated, including DPPH (2,2-diphenyl-1-picrylhydrazyl), ABTS (2,2-azinobis (3-ethylbenzothiazoline-6-sulfonic acid)) radical scavenging activity, and ferric reducing antioxidant power (FRAP), along with α-amylase/α-glucosidase inhibitory effects. Twenty-five phenolics were identified, including L-DOPA (11.96–17.93 mg/g, >70% of total content), seven phenolic acids, and seventeen flavonoids. L-DOPA showed potent enzyme inhibition (IC_50_ values of 22.45 μM for α-amylase and 16.66 μM for α-glucosidase) but demonstrated limited antioxidant effects. Lincan 13 (Gansu) exhibited the strongest antioxidant activity (DPPH, 16.32 μmol trolox/g; ABTS, 5.85 μmol trolox/g; FRAP, 21.38 mmol Fe^2+^/g), which correlated with it having the highest flavonoid content (40.51 mg rutin/g), while Yican 4 (Yunnan) showed the strongest α-amylase inhibition (43.33%). Correlation analysis confirmed flavonoids as the primary antioxidants, and principal component analysis (PCA) revealed geographical trends (e.g., Jiangsu varieties were particularly phenolic-rich). These findings highlight faba beans’ potential as functional foods and guide genotype selection in targeted breeding programs aimed at enhancing health benefits.

## Simple Summary

Faba beans are rich in protein, dietary fiber, vitamins, and minerals. Crucially, they contain significant levels of phytochemicals, particularly phenolic compounds. This study evaluated 29 Chinese faba bean varieties, revealing significant differences in their phytochemical profiles, antioxidant capacity, and enzyme inhibitory effects. Key findings highlight that flavonoids are the primary contributors to antioxidant activity, while L-DOPA, despite its weaker antioxidative properties, shows strong potential for managing blood glucose levels by inhibiting carbohydrate-digesting enzymes (α-amylase and α-glucosidase). These results underscore the potential of faba beans as functional foods tailored to oxidative stress prevention and diabetes management. The established phytochemical markers guide development of cultivars with enhanced nutritional functionality through targeted selection. By bridging the gap between traditional crops and modern health demands, this research supports the growing interest in plant-based solutions for chronic diseases and promotes sustainable dietary strategies.

## 1. Introduction

Faba bean (*Vicia faba* L.), also known as broad bean, is a globally significant legume crop (family Fabaceae) widely consumed due to its high nutritional value, including proteins, dietary fiber, vitamins, minerals, and bioactive phytochemicals that are beneficial to human health [1]. Among phytochemicals, phenolic compounds, especially flavonoids and phenolic acids, are of particular interest due to their diverse biological activities, including antioxidant, anti-inflammatory, antidiabetic, and neuroprotective effects [1,2,3,4]. Notably, faba beans are among the richest natural sources of L-3,4-dihydroxyphenylalanine (L-DOPA), a phenolic amino acid and precursor to neurotransmitter dopamine, which has significant pharmacological value in Parkinson’s disease treatment [5,6]. Beyond its neurological benefits, L-DOPA and other phenolic compounds in faba beans may contribute to antioxidant and enzyme inhibitory activities, particularly against starch-digesting enzymes such as α-amylase and α-glucosidase, key targets in managing postprandial hyperglycemia [7,8].

The phenolic composition and bioactivity vary significantly among different faba bean varieties, depending on genetic and environmental factors [9,10]. While some cultivars contain more flavonoids, others are particularly rich in L-DOPA, which may affect their functional properties [11]. For instance, dark-seeded varieties typically contain higher phenolic levels than their lighter-colored counterparts. Furthermore, processing methods including soaking, fermentation, and thermal treatment can significantly alter phenolic bioavailability and bioactivity [12]. As plant-based functional foods gain prominence in metabolic disease management, elucidating varietal differences becomes critical for faba bean applications in therapeutic diets and functional food formulations.

Phytochemical and antioxidant characterization represents a major factor in cultivar selection for breeding programs [11]. Recent advances in analytical techniques, particularly high-performance liquid chromatography (HPLC) and ultrahigh-performance liquid chromatography with quadrupole time-of-flight mass spectrometry (UPLC-QTOF-MS), have revolutionized the precise identification and quantification of phenolic compounds in plant matrices [13,14]. The integration of analytical methods with bioassays of antioxidant capacity and enzyme inhibition helps to evaluate potential health benefits of faba bean phenolics [15]. The current research confirms that phenolic-rich legume extracts exhibit significant free radical scavenging activity while also delaying carbohydrate digestion through α-amylase and α-glucosidase inhibition, offering effective modulation of postprandial blood glucose levels [16,17,18]. However, most studies have focused on general phenolic profiles or antioxidant activities without establishing a clear link between specific bioactive compounds, particularly L-DOPA, and carbohydrate-digesting enzyme inhibitory effects. Moreover, comparative analyses of varietal differences in L-DOPA content and its contribution to antidiabetic potential remain limited.

Despite extensive research on faba bean phenolics, comprehensive comparative analyses of Chinese faba bean varieties, which represent significant yet underexplored genetic and geographical diversity, are lacking. This study systematically investigated phenolic composition, antioxidant capacity, and starch-digesting enzyme inhibition across 29 distinct faba bean varieties from China, with a targeted emphasis on L-DOPA’s role. By integrating advanced UPLC-QTOF-MS quantification with bioactivity assays, we demonstrated how variety-specific phenolic profiles, rather than just total phenolic/flavonoid content, influence functional properties. These findings could guide the selection of optimal cultivars for breeding programs and functional food development aimed at oxidative stress prevention and metabolic disorder management.

## 2. Materials and Methods

### 2.1. Chemical and Reagents

Folin–Ciocalteu reagent was obtained from Shanghai Lianmai Bioengineering Co., Ltd. (Shanghai, China). Ferrozine and 2,2-azinobis (3-ethylbenzothiazoline-6-sulfonic acid) diammonium salt (ABTS) were bought from Thermo Fisher Scientific (Waltham, MA, USA). Trolox (6-hydroxy-2,5,7,8-tetramethylchromate-2-carboxylic acid) was sourced from Acros Organics (Morris Plains, NJ, USA). Two enzymes (α-amylase and α-glucosidase), biochemical reagents including acarbose, 3,5-dinitrosalicylic acid (DNS), *p*-nitrophenyl-α-D-glucopyranoside (pNPG), 2,2-diphenyl-1-picrylhydrazyl (DPPH), and 2,4,6-tris (2-pyridyl)-S-triazine, along with nine HPLC-grade standards (purity ≥ 98%), including L-DOPA, gallic acid, protocatechuic acid, catechin, gallocatechin, epigallocatechin, procyanidin B2, rutin (quercetin-3-*O*-rutinoside), and kaempferol-3-*O*-rutinoside, were acquired from Yuanye Chemical Ltd. (Shanghai, China). The HPLC solvent formic acid was purchased from Sigma-Aldrich (St. Louis, MO, USA), while acetonitrile was purchased from TEDIA (Fairfield, OH, USA). All the other chemicals and reagents used were of analytical grade from Nanjing Chemical Reagent Co., Ltd. (Nanjing, China).

### 2.2. Plant Materials and Extraction

Twenty-nine faba bean varieties were obtained from the Institute of Industrial Crops, Jiangsu Academy of Agricultural Sciences (Nanjing, China), collected from ten regions across China (details in Table 1). Dried bean seeds were ground with a DFT-100A high-speed grinder (Lin Da Machinery Co., Ltd., Wenling, China) and sieved through a 20-mesh sieve to obtain homogenized powder. Extracts were prepared from 1 g of dried powder in three sequential aliquots using a total of 25 mL of 85% methanol containing 0.5% formic acid according to a previous method [19]. All the faba bean extracts (FBEs) were stored at 4 °C until further analysis.

**Figure 1:**
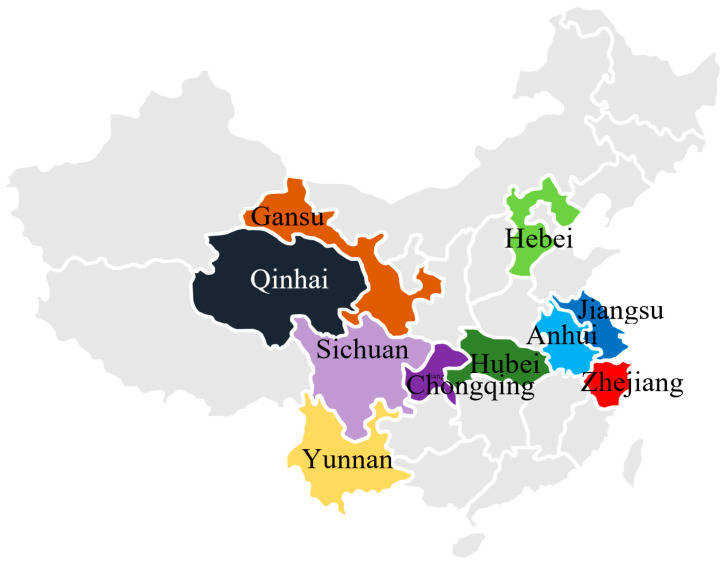
The geographical map displaying the origin of the 29 faba bean varieties.

### 2.3. Determination of Total Phenolic Content

Total phenolic content (TPC) was determined using the modified Folin–Ciocalteu colorimetric method [20]. Briefly, 0.2 mL of FBE was mixed with 2 mL of 0.5 M Folin–Ciocalteu reagent after appropriate dilution. The reaction mixture was vortexed and incubated at room temperature (RT) for 4 min, followed by the addition of 2 mL of 75 g/L Na_2_CO_3_. After 2 h of dark incubation at RT, absorbance was recorded at 760 nm using a spectrophotometer (Thermo Fisher Scientific, Waltham, MA, USA). A calibration curve was established with various concentrations of gallic acid. Results were expressed as milligram gallic acid equivalents per gram of dry weight of faba beans (mg GAE/g DW).

### 2.4. Determination of Total Flavonoid Content

Total flavonoid content (TFC) was quantified following an optimized sodium nitrite-aluminum nitrate method [21]. Briefly, 2.0 mL of FBE was vortex-mixed with 0.2 mL of 5% NaNO_2_ and incubated for 6 min at RT. Subsequently, 0.2 mL of 10% AlCl_3_·6H_2_O was added, followed by a 5 min reaction. The mixture was then neutralized with 1 mL of 1 M NaOH, then incubated for 15 min. Absorbance at 510 nm was measured using a spectrophotometer (Thermo Fisher Scientific, Waltham, MA, USA). A calibration curve was established with rutin standards. The TFC values were expressed as rutin equivalents per gram dry weight (mg RE/g DW).

### 2.5. In Vitro Antioxidant Capacity

The antioxidant capacity of FBEs from 29 faba bean varieties and their major constituent L-DOPA was assessed via three standardized assays, including DPPH radical scavenging activity, ABTS radical cation scavenging activity, and ferric reducing antioxidant power (FRAP) [22]. All colorimetric measurements were performed using the spectrophotometer (Thermo Fisher Scientific, Waltham, MA, USA). The absorbance was measured at 517 nm for DPPH, 734 nm for ABTS, and 593 nm for FRAP, respectively. Ascorbic acid (vitamin C) was used as the positive control. The DPPH and ABTS values were both expressed as Trolox equivalents (µmol TE/g DW), while FRAP values were expressed as ferrous iron (Fe^2+^) equivalents (mmol FE/g DW).

### 2.6. In Vitro Enzyme Inhibitory Effects

The α-amylase and α-glucosidase inhibitory activities of FBEs from 29 faba bean varieties and pure L-DOPA were evaluated according to a previous report [23]. For the α-amylase inhibition assay, 50 µL of each sample was mixed with 50 µL of α-amylase solution (0.1 mg/mL) and incubated at 37 °C for 10 min. The reaction was initiated by adding 100 µL of 1% starch solution, followed by another 10 min incubation at 37 °C. The reaction was terminated by adding 400 µL of DNS reagent, and the mixture was heated at 100 °C for 10 min before measuring absorbance at 540 nm. The α-glucosidase inhibition assay followed an analogous protocol, replacing α-amylase with α-glucosidase and substituting starch substrate with 6 mM pNPG. After 30 min incubation, the reaction was stopped with 500 µL of 0.1 M Na_2_CO_3_ solution, and absorbance was read at 400 nm. Phosphate-buffered saline (PBS) served as the negative control (without sample) and control blank (without enzyme), while acarbose was used as the positive control. For both assays, the inhibition rate was calculated with the following equation:

$$\mathrm{Inhibition}\;\mathrm{percentage}\;(\%)\;=\;\left(1-\frac{A1-A2}{A3-A4}\right)\times 100\%$$

where A1, A2, A3, and A4 represent the absorbance values of the sample reaction, negative control, control reaction, and control blank, respectively.

### 2.7. UPLC-QTOF-MS Analysis

Chromatographic profiling was performed with an Agilent 6538 UHD Q-TOF mass spectrometer (Agilent Technologies, Santa Clara, CA, USA) coupled with an Agilent 1290 Infinity UPLC system via electrospray ionization (ESI) in both positive and negative polarity modes. Separation was achieved on a Waters HSS T3 column (2.1 × 150 mm, 3.5 μm; Waters Corporation, Milford, MA, USA) [24]. The mobile phase consisted of phase A (acetonitrile) and phase B (0.1% formic acid), at a flow rate of 0.4 mL/min with an injection volume of 3 μL. The gradient elution program for phase B was as follows: 95% (0–2 min), 95–60% (2–10 min), 60–0% (10–40 min), 0% (40–43 min), 0–95% (43–43.1 min), and 95% (43.1–48 min). MS detection was performed in full-scan mode (*m*/*z* 100–1000) with high-resolution MS/MS for fragmentation. Data were processed by matching the acquired spectra against the SCIEX TML (Targeted Metabolite Library) database for compound identification.

### 2.8. HPLC Analysis

The HPLC fingerprint analysis was performed using an Agilent 1290 Infinity II HPLC system (Agilent Technologies, Waldbronn, Germany) with an Agilent Poroshell 120 EC-C18 column (4.6 mm × 250 mm, 4 μm). The mobile phase consisted of phase A (0.1% formic acid) and phase B (acetonitrile). According to a previous report [25], the gradient elution program for phase B was as follows: 5% (0–2 min), 5–45% (2–25 min), 45–95% (25–40 min), 95% (40–45 min), 95–5% (45–46 min), and 5% (46–50 min). The injection volume was 10 μL with a flow rate of 1 mL/min, while the column temperature was maintained at 30 °C. Chromatograms were acquired using a diode array detector (DAD) at 280 nm. Nine major phenolics were quantified using their relative standard curves (L-DOPA, *y* = 12.56*x* − 257.52, *R*^2^ = 0.9988; gallic acid, *y =* 45.767*x −* 70.033, *R*^2^ = 0.9968; protocatechuic acid, *y* = 50.671*x* + 690.98, *R*^2^ = 0.9968; gallocatechin, *y* = 7.6211*x* − 77.895, *R*^2^ = 0.9997; epigallocatechin, *y* = 15.932*x* − 41.637, *R*^2^ = 0.9978; catechin, *y* = 20.612*x* − 36.328, *R*^2^ = 0.9997; procyanidin B2, *y* = 61.919*x* − 94.513, *R*^2^ = 0.9965; rutin, *y* = 67.957*x* + 34.355, *R*^2^ = 0.9998; kaempferol-3-*O*-rutinoside, *y* = 77.482*x* + 53.036, *R*^2^ = 0.9998). The limits of detection (LOD) and quantification (LOQ) for the analytical standards ranged from 0.05 to 0.79 µg/mL and 0.17 to 2.61 µg/mL, respectively, with recoveries of 90–98%. The results were expressed as milligrams of each phenolic compound per 100 g dry weight, i.e., mg/100 g DW, except for L-DOPA (mg/g DW).

### 2.9. Statistics Analysis

All experimental procedures were performed in triplicate with data presented as mean ± standard deviation (SD). Regression equation slopes were calculated by Microsoft Excel 2016. Statistical analyses and graphical representations were generated using GraphPad Prism 9.5 (GraphPad Software, Inc., San Diego, CA, USA). Person correlation coefficients (*R*) and *p*-values were calculated using the Social Science Statistics online platform (https://www.socscistatistics.com/tests/pearson/, accessed on 26 May 2025). Principal component analysis (PCA) was performed using JMP Pro 10 software (SAS Institute Inc., Cary, NC, USA). Significant differences (*p* < 0.05) were determined by one-way analysis of variance (ANOVA).

## 3. Results and Discussion

### 3.1. Total Phenolic and Flavonoid Content in 29 Faba Bean Varieties

Phenolic compounds, particularly flavonoids, represent a major class of phytochemicals renowned for their diverse bioactivities and crucial roles in modulating biochemical and nutritional properties in plants [26]. Faba beans serve as an exceptionally rich source of these functional components, with well-documented high concentrations in seeds, leaves, and immature pods [3,11,27]. Supporting this profile, our analysis of seeds from 29 distinct faba bean varieties, sourced from diverse agroecological regions across China, revealed remarkable phenolic abundance. Comprehensive quantification demonstrated significant variation in both total phenolic content (TPC) and total flavonoid content (TFC) among the evaluated genotypes, as detailed in Table 1.

The TPC values showed limited variation among the 29 samples, ranging from 10.46 ± 0.99 mg GAE/g DW (Baihuahudou, Yunnan, China) to 18.07 ± 0.26 mg GAE/g DW (Zhecan 1, Zhejiang), with an overall mean of 13.77 ± 2.07 mg GAE/g DW. Except for the top-performing Zhecan 1 variety from Zhejiang Province, the other six varieties exceeded 15 mg GAE/g DW, including Zhongjiangchangxiu (Jiangsu), Lincan 13 (Gansu), 2016-831 (Jiangsu), Dingxuancan 3 (Jiangsu), Jizhangcan 5 (Hebei), and Edou 1103 (Hubei). Geographically, Jiangsu Province accounted for three of these high-TPC varieties, while single representatives came from Gansu, Hebei, and Hubei. The TFC values varied significantly across varieties, ranging from 14.65 ± 0.59 to 40.51 ± 0.39 mg RE/g DW, with a mean of 24.21 ± 5.94 mg RE/g DW. Lincan 13 (Gansu) exhibited the highest TFC, followed by two Jiangsu varieties Dingxuancan 3 (38.10 ± 0.55 mg RE/g DW) and Tongcanxian 20 (33.49 ± 0.50 mg RE/g DW). Conversely, three Yunnan varieties, Fengdou 11, Yican 6, and Chuzao 1, displayed the lowest TFC values (<18 mg RE/g DW). Approximately 66% of varieties fell within the moderate TFC range (20–30 mg RE/g DW), indicating prevalent flavonoid accumulation across most faba bean accessions.

The observed phytochemical diversity among the 29 faba bean varieties resulted from complex gene–environment interactions. Genotypic differences primarily drive phenotypic variation, evidenced by the cultivar-specific accumulation of phenolics that directly modulate organoleptic properties such as color and taste [28,29]. Valente et al. confirmed this genetic basis, demonstrating significant divergence in tannins, flavonoids, and proanthocyanidins among five international faba bean genotypes from Turkey, Italy, Argentina, the USA, and Hungary [30]. Genome-wide analyses further revealed geographically structured variation: the faba bean germplasm comprises three genetically distinct subpopulations shaped by adaptive divergence, with molecular markers differentiating northern and southern accessions accounting for substantial agronomic trait variance [10]. Environmental factors, including climate, soil composition, altitude, and sulfur fertilization, concurrently regulate phenolic biosynthesis [31,32]. This interplay was exemplified by Jiangsu’s highly phenolic varieties (e.g., Zhongjiangchangxiu), which benefited from fertile soils and humidity, contrasting with Gansu’s flavonoid-rich Lincan 13 variety, where high-altitude stressors like UV exposure stimulated secondary metabolite production [33]. Zeng et al. demonstrated that *Vaccinium* ‘Brightwell’ blueberries cultivated at higher altitudes exhibited elevated sweetness, which promoted substrate availability for anthocyanin biosynthesis and consequently enhanced anthocyanin accumulation [34]. Liu et al. reported notable variations in bioactive compound profiles and antioxidant capacity across distinct jujube cultivars (e.g., Yuanlingzao, Dongzao, *Ziziphus jujuba* Mill.), demonstrating the substantial influence of genetic background on nutritional quality [35]. Phenolic content in food products are determined by a complex interplay of factors, including genetic characteristics (cultivar or variety), agronomic practices (irrigation, fertilization, and pest/disease control), harvest maturity, storage conditions, and cultivation climate [36]. Thus, region-specific chemotypes emerge from synergistic genotype × environment interactions, where localized selective pressures fine-tune genetic potential into adaptive phytochemical profiles.

### 3.2. Preliminary Identification of Phenolic Compounds in Faba Beans

The novel analytical technique UPLC-QTOF-MS integrates ultra-fast chromatographic separation with high-resolution exact mass measurement and diagnostic fragmentation, enabling robust structural characterization of diverse phenolic compounds. In this study, 25 phenolics were tentatively identified by systematically matching retention times (t_R_), elution-order patterns, exact masses, and characteristic MS/MS fragment ions against in-house standards, commercial libraries, and literature-derived spectral databases [3,30]. Detailed characterization data, including chemical formulas, theoretical/observed mass-to-charge ratios (*m*/*z*), and mass errors (ppm) in both positive/negative ESI modes, are comprehensively presented in Table 2.

The identification of L-DOPA ([M + H]^+^, *m*/*z* 198.0761) in the crude faba bean extract was confirmed by both exact mass matching (0 ppm error) and the presence of a diagnostic fragment ion at *m*/*z* 77.0419. This fragment, characteristic of aromatic ring cleavage in benzene derivatives, provides additional structural validation beyond exact mass alone. Notably, faba beans represent one of the richest natural plant sources of L-DOPA, which is consistent with its detection here. As a major bioactive compound in *V. faba*, L-DOPA has been consistently identified across multiple plant tissues, including leaves, seedlings, immature pods, and grains [6,11].

Seven phenolic acids were identified in the FBE, including gallic acid, cinnamic acid, both coumaric acid and *p*-coumaric acid, *p*-hydroxybenzoic acid, protocatechuic acid, and ferulic acid. The detection of coumaric acid in positive ion mode, cinnamic acid in both positive and negative ion modes, and the majority of phenolic acids in negative ion mode highlights the necessity of employing complementary ionization techniques for comprehensive phenolic acid profiling. This dual-polarity approach enhances detection sensitivity and reliability across different compound classes. The specific phenolic acids identified here (e.g., protocatechuic acid, *p*-coumaric acid, ferulic acid) were consistent with those previously reported in faba beans. Notably, Valente et al. demonstrated that these acids exist in both free and esterified forms within faba beans [30]. This distribution between free and conjugated fractions is crucial as it significantly influences their bioavailability and potential bioactivity.

In addition, a total of 17 flavonoids and/or their derivatives were identified, including four flavanols (gallocatechin, epigallocatechin, catechin, and epicatechin), one anthocyanin (cyanidin-3-*O*-glucoside), one proanthocyanidin (procyanidin B2), two flavonols (myricetin and quercetin), and nine flavonol glycosides (quercetin-3-*O*-glucoside, quercetin-3-*O*-rutinoside (rutin), quercetin-3-beta-sophoroside, quercetin-3-*O*-rhamnoside (quercitrin), quercetin-7-rhamnoside, kaempferol-3-*O*-rutinoside, Kaempferol-3,7-*L*-dirhamnoside (Kaempferitrin), myricetin-3-*O*-rhamnoside (Myricitrin), and isorhamnetin-3-*O*-neohespeidoside). This extensive flavonoid profile, particularly the predominance of flavonol glycosides, not only confirmed but also significantly expanded upon previous characterizations of faba bean phenolics. Previous studies [3,30,37] have identified flavonol glycosides, proanthocyanidins (e.g., prodelphinidin), flavanols (such as catechin and epicatechin), and flavone glycosides across various genotypes. The combined detection of diverse phenolic acids and a broad spectrum of flavonoids, including complex glycosides and condensed tannins (e.g., procyanidin B2), underscores the metabolic versatility of faba beans in synthesizing phenolic compounds. This rich array of phenolics contributes substantially to the plant’s defense mechanisms and its nutritional value as a dietary source of bioactive phytochemicals [4].

### 3.3. Quantification Analysis of Phenolic Compounds in 29 Faba Bean Varieties

**Figure 2:**
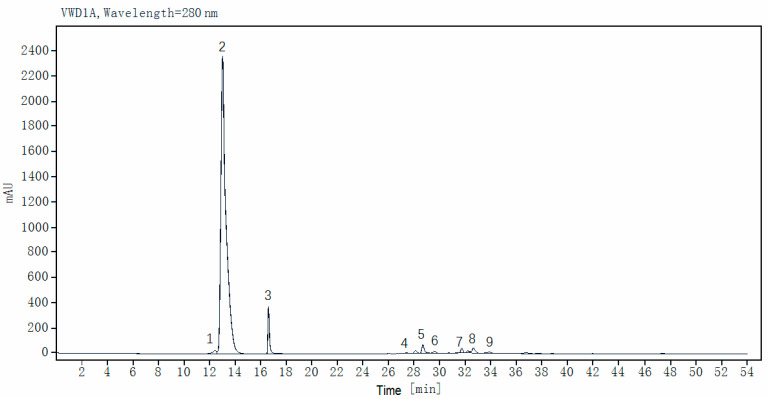
Typical HPLC chromatogram of faba bean extracts at 280 nm. Peaks: 1, gallic acid; 2, L-DOPA (L-3,4-dihydroxyphenylalanine); 3, protocatechuic acid; 4, gallocatechin; 5, epigallocatechin; 6, catechin; 7, procyanidin B2; 8, rutin (quercetin-3-*O*-rutinoside); 9, kaempferol-3-*O*-rutinoside.

The eight major phenolic components in 29 faba bean varieties were further quantified using reference standards by HPLC analysis (Table 3; a representative HPLC profile is shown in Figure 2). L-DOPA emerged as the overwhelmingly dominant phenolic metabolite, accounting for >70% of the total quantified phenolics in most varieties, with its contents ranging from 11.96 ± 0.08 mg/g DW (Tongcanxian 21) to 17.93 ± 0.21 mg/g DW (Zhongjiangchangxiu), averaging 15.12 mg/g DW. Notably, the L-DOPA levels were consistently ~100-fold higher than those of the other quantified phenolics, reinforcing its unparalleled quantitative significance within the faba bean phenolic profile. This profound quantitative dominance of L-DOPA (~100-fold higher than other major phenolics and isoflavones such as daidzin/genistein derivatives) was observed across different tissues of faba bean genotypes, including grains, leaves, and immature pods [11]. The exceptionally high concentrations quantified here solidified faba beans’ status as the best natural plant source of L-DOPA. This metabolic prominence conferred their significant nutritional value and potent therapeutic potential, particularly for neurological health [5,38].

A notable genotypic and regional variability in L-DOPA accumulation was observed. The highest (Zhongjiangchangxiu and Dingxuancan 3) and lowest (Tongcanxian 21 and Tongcanxian 20) L-DOPA-producing varieties all originated from Jiangsu Province, highlighting the complex interplay of genetic background and localized environmental factors (e.g., microclimate, soil composition) in modulating L-DOPA biosynthesis. Extensive research confirmed that L-DOPA levels in faba beans are strongly influenced by both cultivar genetics and key environmental variables, including geographical location, temperature regimes, and harvest timing [39,40,41].

Beyond the overwhelming dominance of L-DOPA, quantitative analysis revealed distinct hierarchies and significant variability among other secondary phenolic metabolites. In the phenolic acid subclass, protocatechuic acid (7.21 ± 0.37 to 16.37 ± 0.19 mg/100 g DW) and gallic acid (2.11 ± 0.01 to 4.60 ± 0.02 mg/100 g DW) emerged as the most abundant, highlighting their metabolic prominence. Flavanols exhibited distinct concentration patterns, with epigallocatechin levels approximately 10-fold higher than those of gallocatechin and catechin, indicating preferential biosynthesis or stabilization of this isomer. Proanthocyanidin accumulation showed striking inter-varietal differences. Procyanidin B2 was highly concentrated in varieties like Jingdou 5 (5.47 mg/100 g DW) and Dingxuancan 3 (3.96 mg/100 g DW), while it had negligible levels (<0.5 mg/100 g DW) in eight others (e.g., Chenghu 21, Chenghudabai, Edou 1103, Edou 1208, Fengdou 11, Fengdou 28, Yican 4, and Yican 6). Such extreme variability suggests strong genetic control over proanthocyanidin production. Despite their structural diversity, flavonol glycosides occurred at relatively low concentrations. Rutin levels ranged from 1.57 ± 0.01 to 8.31 ± 0.01 mg/100 g DW, while kaempferol-3-*O*-rutinoside varied from 0.17 ± 0.00 to 3.35 ± 0.01 mg/100 g DW, demonstrating that compositional diversity does not correlate with abundance.

Notably, geographical origin showed a significant correlation with phenolic profiles [3]. Varieties from Jiangsu Province (e.g., Dingxuancan 3, Tongcanxian 20) consistently accumulated higher levels of L-DOPA while also exhibiting elevated concentrations of specific flavonoids, a pattern that contrasted sharply with the phenolic distribution observed in Yunnan varieties. These findings underscored the profound influence of regional growing conditions (e.g., climate, soil composition, and agronomic practices) interacting with genetic background [10,31,32] in shaping faba beans’ quantitative phenolic composition, extending beyond the well-characterized effects on L-DOPA accumulation.

### 3.4. In Vitro Antioxidant Capacity of 29 Faba Bean Varieties

The antioxidant capacity of faba beans was comprehensively evaluated using three established assays, DPPH, ABTS, and FRAP, each targeting distinct mechanisms. The DPPH assay quantified lipophilic free radical scavenging activity, reflecting hydrogen-donating capacity [21]. The ABTS assay measured broad-spectrum antioxidant activity against cationic radicals in both aqueous and organic phases, while the FRAP assay determined reducing power through Fe^3+^ to Fe^2+^ transformation [42]. Significant variability was observed among the 29 varieties (Table 1), likely attributable to differences in phenolic composition.

The DPPH radical scavenging activity exhibited a 2.3-fold variation among varieties (7.24 ± 0.35 to 16.32 ± 0.31 μmol TE/g DW), with Lincan 13 (Gansu origin) showing the highest activity. The ABTS assay revealed an antioxidant capacity range of 2.08 ± 0.06 to 5.85 ± 0.20 μmol TE/g DW, where Lincan 13 again demonstrated superior performance. The FRAP values showed the widest variation (11.82 ± 0.36 to 21.38 ± 0.68 mmol FE/g DW) among all tested methods. Remarkably, Lincan 13 from Gansu consistently ranked highest across all assays, correlating with its elevated TFC (40.51 ± 0.39 mg RE/g DW). Two Jiangsu varieties (Dingxuancan 3 and Tongcanxian 20) also exhibited exceptional antioxidant capacity, ranking among the top three performers. In contrast, Yunnan varieties generally displayed lower antioxidant activity, consistent with their reduced phenolic and flavonoid levels.

These findings demonstrated significant varietal differences in faba bean antioxidant capacity, which correlated strongly with cultivar-specific phenolic profiles influenced by geographical origin [3]. Consistent with the genetic regulation of phytochemical composition observed across plant species [37], this inherent phenolic variability represented the principal determinant of antioxidant properties. The comprehensive multi-assay evaluation establishes robust structure–activity relationships between specific phenolic compounds (particularly hydroxyl/carboxyl-rich phenolic acids and B-ring-substituted flavonoids) and observed bioactivity gradients across genotypes [43,44]. Together, these insights provide a scientific foundation for targeted the breeding of antioxidant-rich faba bean cultivars optimized for nutraceutical and functional food applications.

### 3.5. In Vitro Enzyme Inhibitory Effects of 29 Faba Bean Varieties

α-Amylase and α-glucosidase are key enzymes in carbohydrate digestion. α-Amylase hydrolyzes starch into oligosaccharides, while α-glucosidase further breaks down oligosaccharides into absorbable monosaccharides that enter systemic circulation, elevating postprandial blood glucose levels [45]. Competitive inhibitors of these enzymes bind to their active sites, delaying carbohydrate digestion and glucose absorption. Therefore, these inhibitors serve as first-line therapeutics for type 2 diabetes management by attenuating postprandial glycemic spikes [46]. Here, the inhibition rates of α-amylase and α-glucosidase varied widely among the faba bean varieties (Table 1).

The α-amylase inhibition rates ranged from 15.17 ± 0.40% (Lican 3, Zhejiang) to 43.33 ± 0.90% (Yican 4, Yunnan), averaging 29.73 ± 7.84% across all varieties. Yican 4 (Yunnan) and Chenghu 21 (Sichuan, 43.24 ± 1.20%) exhibited the strongest α-amylase inhibitory effects, surpassing most varieties by >10%. For α-glucosidase inhibition, values ranged from 4.26 ± 0.70% (Dingxuancan 3, Jiangsu) to 22.05 ± 0.30% (Lincan 13, Gansu), with a mean value of 10.87 ± 4.31%. Lincan 13 (Gansu) and Lican 7 (Zhejiang, 19.54 ± 0.90%) demonstrated the highest α-glucosidase inhibitory activity, indicating their particular potential for postprandial glucose modulation. Notably, Lincan 13 (Gansu) showed dual efficacy, ranking highly in both antioxidant capacity and enzyme inhibition. In contrast, Dingxuancan 3 (Jiangsu), despite its strong antioxidant activity, displayed the lowest α-glucosidase inhibition. This apparent disparity suggests that different bioactive compounds may mediate the antioxidant and antidiabetic properties of faba beans.

Fava bean flour (composed primarily of carbohydrates and proteins) and its protein hydrolyzates exhibited inhibitory effects on α-amylase and/or α-glucosidase. Processing methods such as toasting and storage were found to enhance this inhibitory activity, thereby modulating glycemic response by limiting carbohydrate digestion [47,48]. Our study similarly observed moderate α-amylase and α-glucosidase inhibition in phenolic extracts from faba beans, consistent with previous findings [49]. Randhir et al. reported grain sprouts’ and seedlings’ α-amylase and α-glucosidase inhibitory activity and found that thermal processing of grain sprouts generally enhanced α-glucosidase inhibitory activity in wheat, buckwheat, and oats but reduced it in corn sprouts, while α-amylase inhibition increased only in buckwheat and oats, correlating with thermally modified phenolic profiles [50].

### 3.6. Correlation Among Phenolics and Bioactivities

**Figure 3:**
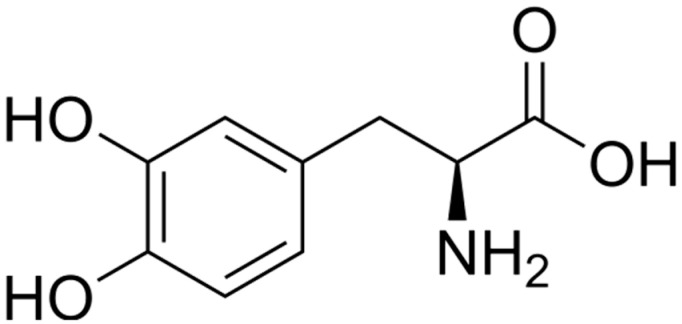
Chemical structure of L-DOPA (L-3,4-dihydroxyphenylalanine).

As the predominant phenolic compound in faba beans, L-DOPA likely serves as a key contributor to their bioactivity. While pure L-DOPA demonstrated moderate antioxidant capacity across all three assays (DPPH, ABTS, and FRAP), its activity remained significantly lower than the positive control ascorbic acid (Table 4). Although L-DOPA contains two phenolic hydroxyl groups (Figure 3) as a dopamine precursor amino acid, its direct antioxidant capacity is substantially limited compared to classical antioxidants [51]. Nevertheless, synergistic interactions with gallic acid, rutin, and proanthocyanidins might potentiate its bioactivity, as demonstrated in combined antioxidant assays [6]. In contrast, L-DOPA exhibited potent inhibition of carbohydrate-digesting enzymes, with IC_50_ values of 22.45 μmol/L for α-amylase and 16.66 μmol/L for α-glucosidase, though still less potent than the positive control acarbose. Beyond its established neuroprotective effects, accumulating evidence suggests that L-DOPA may function as a potential antidiabetic agent. This is supported by studies of L-DOPA-rich faba bean sprouts, which have demonstrated significant hypoglycemic effects, antidiabetic activity, and attenuation of diabetes-related complications [52].

**Figure 4:**
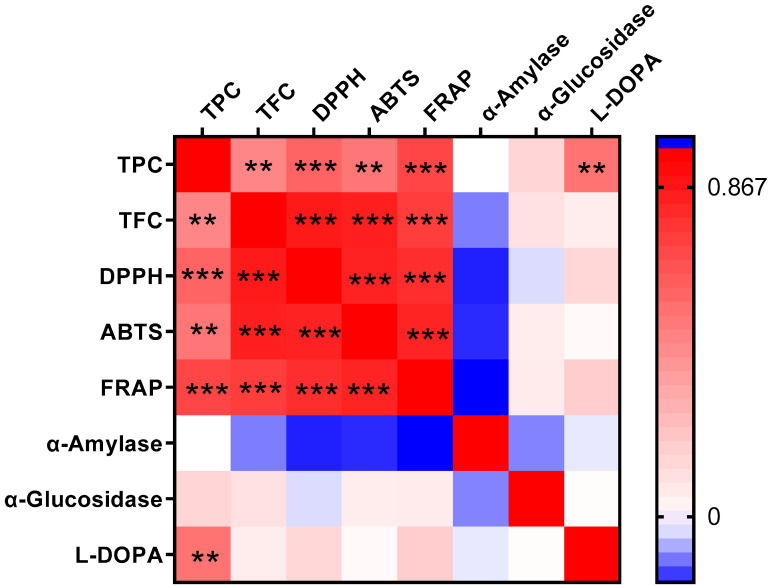
Heatmap of the Person correlation analysis among the eight indicators of the 29 faba bean varieties. TPC, total phenol content; TFC, total flavonoid content; DPPH, DPPH radical scavenging capacity; ABTS, ABTS radical cation scavenging activity; FRAP, ferric reducing antioxidant power; α-Amylase, α-amylase inhibitory effect; α-Glucosidase, α-glucosidase inhibitory effect; L-DOPA, L-3,4-dihydroxyphenylalanine content. Red represents a positive correlation, while blue represents a negative correlation. Differences were considered significant at ** *p* < 0.01 and *** *p* < 0.001.

Pearson correlation analysis of eight biochemical indicators across 29 faba bean varieties (Figure 4) revealed highly significant (*p* < 0.001) positive correlations among antioxidant capacity measures and total flavonoid content. Specifically, significant positive correlations existed between each pair of antioxidant indexes (TFC vs. DPPH, *R* = 0.8707; TFC vs. ABTS, *R* = 0.8584; TFC vs. FRAP, *R* = 0.7500; DPPH vs. ABTS, *R* = 0.8447; DPPH vs. FRAP, *R* = 0.8013; ABTS vs. FRAP, *R* = 0.8390). These results indicated that flavonoids served as the primary antioxidants in these faba beans. Kwon et al. also found a positive correlation between the DPPH radical scavenging activity and the flavone di- and tri-glycosides [3]. Total phenolic content showed moderate positive correlations with both antioxidant measures (DPPH, *R* = 0.6061; ABTS, *R* = 0.5411; FRAP, *R* = 0.7182) and L-DOPA levels (*R* = 0.5497), consistent with previous reports [30,53]. Notably, neither α-amylase nor α-glucosidase inhibition correlated significantly with phenolic/flavonoid content, antioxidant activity, or L-DOPA levels. The observed negative correlations between α-amylase inhibition and other indicators suggest that its regulation involves alternative factors, potentially including polysaccharides and proteins [47,48].

**Figure 5:**
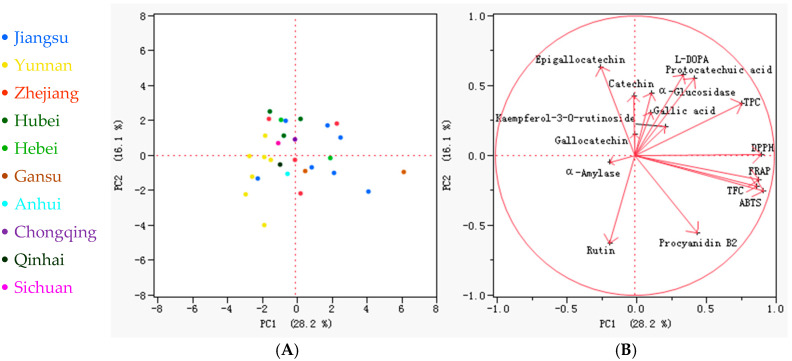
The biplots and loading plots of the principal component analysis (PCA), involving the 16 indicators of the 29 faba bean varieties. (**A**) PCA scores of the faba bean samples. The different colors represent faba bean varieties from different places of origin; (**B**) PCA loadings of the 16 parameters, including TPC (total phenol content), TFC (total flavonoid content), DPPH (DPPH radical scavenging capacity), ABTS (ABTS radical cation scavenging activity), FRAP (ferric reducing antioxidant power), α-Amylase (α-amylase inhibitory effect), α-Glucosidase (α-glucosidase inhibitory effect), L-DOPA (L-3,4-dihydroxyphenylalanine), gallic acid, protocatechuic acid, gallocatechin, epigallocatechin, catechin, procyanidin B2, rutin, and kaempferol-3-*O*-rutinoside contents.

The principal component analysis (PCA) of 29 faba bean varieties across 16 phytochemical parameters (including phenolics, antioxidant activity, and enzyme inhibition) revealed distinct metabolic variation patterns (Figure 5). The first two principal components accounted for 44.3% of the total variance (PC1: 28.2%; PC2: 16.1%). The score plot (Figure 5A) (Figure 5A) demonstrated partial geographical clustering. Jiangsu varieties were predominantly distributed along the positive PC1 axis, whereas Yunnan varieties were clustered negatively along PC1. Lincan 13 emerged as a distinct outlier (PC1 > 0, PC2 > 0), characterized by exceptionally high L-DOPA and antioxidant capacity. The loading plot (Figure 5B) revealed that PC1 loadings highlighted strong positive associations for TFC, TPC, antioxidant assays (DPPH, ABTS, FRAP), and procyanidin B2, while α-amylase inhibition loaded negatively, indicating its independence from antioxidant traits. Along PC2, L-DOPA, and catechin loaded positively, whereas procyanidin B2 and rutin loaded negatively. Notably, the orthogonal distribution of L-DOPA relative to other phenolics along PC2 confirmed its unique variation pattern, consistent with the correlation results. This metabolic separation suggested that breeding for high-L-DOPA varieties might not necessarily enhance antioxidant potential, but it could specifically improve antidiabetic enzyme inhibition, aligning with previous reports [52,54]. Such specificity supports the development of targeted breeding strategies for either antioxidant-rich or L-DOPA-enriched varieties based on distinct metabolic trade-offs.

## 4. Conclusions

This study provided a comprehensive chemogeographic profiling of the Chinese faba bean, characterizing 29 varieties across 10 provinces to reveal significant geographical and genotypic diversity in phenolic composition, antioxidant capacity, and carbohydrate-digesting enzyme inhibitory effects. Jiangsu varieties emerged as phenolic-rich hubs, while Gansu’s Lincan 13 variety exhibited exceptional flavonoid-driven antioxidant activity. As the predominant phenolic compound in faba beans, L-DOPA was discovered as having a unique dual bioactivity dichotomy– weak antioxidant effects versus potent inhibition of α-amylase and α-glucosidase—suggesting a novel phenolic-mediated glycemic control mechanism independent of classical antioxidants. These findings bridged the gap between traditional crops and modern health demands, positioning faba beans as versatile functional foods tailored to oxidative stress prevention or diabetes intervention (high-L-DOPA varieties). It would be helpful to carry out precision breeding to develop location-specific cultivars and targeted dietary strategies to combat chronic diseases. Future work could employ molecular docking to elucidate the underlying mechanism of L-DOPA’s effects on glucosidase inhibition and validate these geographical patterns through multi-season and multi-location trials.

## Figures and Tables

**Table 1:** Total phenolic content (TPC), total flavonoid content (TFC), antioxidant capacity, and enzyme inhibitory effects of 29 faba bean varieties.

Faba Bean Varieties	Origin ^a^	TPC (mg GAE/g DW)	TFC (mg RE/g DW)	DPPH (µmol TE/g DW)	ABTS (µmol TE/g DW)	FRAP (mmol FE/g DW)	Inhibition Rate of α-Amylase (%)	Inhibition Rate of α-Glucosidase (%)
Baihuahudou	Yunnan	1.05 ± 0.10	2.24 ± 0.02	7.92 ± 0.04	2.15 ± 0.05	11.89 ± 0.16	22.68 ± 0.70	10.71 ± 0.90
Chenghu 21	Sichuan	1.40 ± 0.03	2.04 ± 0.07	8.91 ± 0.56	3.13 ± 0.66	14.66 ± 0.45	43.24 ± 1.20	10.44 ± 0.90
Chenghudabai	Hebei	1.38 ± 0.08	1.92 ± 0.08	8.77 ± 0.39	2.78 ± 0.14	14.59 ± 0.29	31.19 ± 0.50	14.86 ± 0.60
Chuzao 1	Yunnan	1.26 ± 0.01	1.78 ± 0.07	8.42 ± 0.67	2.08 ± 0.06	12.27 ± 0.53	32.62 ± 0.40	9.08 ± 0.10
Cican 1	Zhejiang	1.24 ± 0.01	2.13 ± 0.02	11.47 ± 0.04	3.42 ± 0.27	16.12 ± 0.42	24.89 ± 0.70	8.74 ± 0.50
Dingxuancan 3	Jiangsu	1.62 ± 0.05	3.81 ± 0.06	15.52 ± 0.41	4.76 ± 0.16	18.40 ± 0.31	37.15 ± 1.00	4.26 ± 0.70
Edou 1103	Hubei	1.58 ± 0.02	2.22 ± 0.02	8.20 ± 0.59	3.64 ± 0.41	16.54 ± 0.77	23.53 ± 0.00	8.77 ± 0.20
Edou 1208	Hubei	1.40 ± 0.03	1.80 ± 0.08	8.39 ± 0.52	2.69 ± 0.45	15.03 ± 0.54	39.86 ± 0.80	13.41 ± 0.60
Fengdou 11	Yunnan	1.24 ± 0.06	1.47 ± 0.06	8.82 ± 0.32	2.49 ± 0.23	13.07 ± 0.05	16.00 ± 0.10	9.65 ± 0.40
Fengdou 28	Yunnan	1.36 ± 0.03	1.99 ± 0.04	9.60 ± 0.22	3.17 ± 0.05	13.33 ± 0.46	36.32 ± 0.60	12.16 ± 0.70
Jianlixiacandou	Hubei	1.34 ± 0.06	1.87 ± 0.09	9.51 ± 0.17	2.89 ± 0.05	14.08 ± 0.72	24.17 ± 0.60	6.29 ± 0.20
Jingdou 5	Yunnan	1.12 ± 0.06	2.19 ± 0.07	9.78 ± 0.51	2.95 ± 0.02	11.82 ± 0.36	40.77 ± 0.30	7.23 ± 0.80
Jizhangcan 5	Hebei	1.62 ± 0.01	2.81 ± 0.05	12.21 ± 0.11	3.89 ± 0.15	16.64 ± 0.64	29.81 ± 0.50	9.24 ± 0.10
Lican 3	Zhejiang	1.08 ± 0.09	2.69 ± 0.02	11.56 ± 0.13	4.49 ± 0.16	16.12 ± 0.50	15.17 ± 0.40	7.97 ± 0.20
Lican 7	Zhejiang	1.29 ± 0.08	2.22 ± 0.06	7.50 ± 0.46	3.06 ± 0.08	12.59 ± 0.09	22.78 ± 0.80	19.54 ± 0.90
Lincan 13	Gansu	1.71 ± 0.06	4.05 ± 0.04	16.32 ± 0.31	5.85 ± 0.20	21.38 ± 0.68	21.67 ± 0.20	22.05 ± 0.30
Lincan15	Gansu	1.30 ± 0.03	2.36 ± 0.09	10.08 ± 0.25	3.47 ± 0.17	16.79 ± 0.48	27.79 ± 0.70	8.24 ± 0.00
Qidou 2	Jiangsu	1.25 ± 0.05	2.44 ± 0.01	9.52 ± 0.29	2.94 ± 0.05	12.40 ± 0.55	23.86 ± 0.80	16.05 ± 0.40
Qingcan 25	Qinghai	1.27 ± 0.03	2.09 ± 0.05	9.55 ± 0.12	2.62 ± 0.12	14.41 ± 0.40	23.45 ± 0.70	12.10 ± 0.40
Tongcanxian 20	Jiangsu	1.48 ± 0.07	3.35 ± 0.05	13.34 ± 0.36	4.64 ± 0.04	19.20 ± 0.79	36.83 ± 0.60	9.66 ± 0.50
Tongcanxian 21	Jiangsu	1.14 ± 0.06	2.47 ± 0.06	8.76 ± 0.48	3.12 ± 0.11	12.18 ± 0.54	40.77 ± 0.20	12.09 ± 0.30
Wancan 1	Anhui	1.14 ± 0.08	2.39 ± 0.14	9.92 ± 0.48	4.21 ± 0.24	13.77 ± 0.14	30.21 ± 0.30	6.47 ± 0.10
Yican 4	Yunnan	1.25 ± 0.10	2.16 ± 0.06	7.24 ± 0.35	2.77 ± 0.25	14.00 ± 0.16	43.33 ± 0.90	18.34 ± 0.30
Yican 6	Yunnan	1.38 ± 0.08	1.68 ± 0.04	7.51 ± 0.13	2.93 ± 0.39	14.51 ± 0.42	37.80 ± 0.60	5.68 ± 0.50
Yucan 1	Chongqing	1.44 ± 0.10	2.62 ± 0.11	10.28 ± 0.30	3.17 ± 0.05	13.01 ± 0.64	37.95 ± 1.40	7.84 ± 0.30
Zhecan 1	Zhejiang	1.81 ± 0.03	2.84 ± 0.10	12.18 ± 0.28	4.51 ± 0.23	17.45 ± 0.67	28.42 ± 0.30	17.27 ± 0.70
Zhongjiang changxiu	Jiangsu	1.78 ± 0.02	2.81 ± 0.09	12.36 ± 0.07	3.69 ± 0.08	17.55 ± 0.71	19.82 ± 0.40	8.99 ± 0.50
2016-831	Jiangsu	1.65 ± 0.07	2.32 ± 0.02	13.28 ± 0.27	3.69 ± 0.18	15.91 ± 0.44	33.81 ± 0.30	11.09 ± 0.10
P16-06-3	Jiangsu	1.39 ± 0.01	2.78 ± 0.07	11.70 ± 0.64	4.40 ± 0.03	16.63 ± 0.21	28.29 ± 0.40	8.92 ± 0.20

^a^ A geographical map displaying the origin of the 29 faba bean varieties is shown in Figure 1. DPPH, DPPH radical scavenging activity; ABTS, ABTS radical cation scavenging activity; FRAP, ferric reducing antioxidant power; GAE, gallic acid equivalent; RE, rutin equivalent; TE, Trolox equivalent; FE, Fe^2+^ equivalent.

**Table 2:** Qualitative results for major phenolic components in the faba bean extracts by UPLC-QTOF-MS analysis.

Compound Identification	Mode	t_R_ (min)	Formula	Expected *m*/*z*	Found at *m*/*z*	Error (ppm)	Major Fragment Ions (*m*/*z*)
L-3,4-dihydroxyphenylalanine (L-DOPA)	[M + H]^+^	1.77	C_9_H_11_NO_4_	198.0761	198.0761	0.0	77.0419
**Phenolic acids and derivatives**							
Gallic acid	[M − H]^–^	3.38	C_7_H_6_O_5_	169.0143	169.0143	0.3	125.0239, 79.0218
Cinnamic acid	[M + H]^+^	4.16	C_9_H_8_O_2_	149.0597	149.0598	0.5	77.0424
	[M − H]^–^	4.17	C_9_H_8_O_2_	147.0452	147.0453	0.7	103.0547, 77.0401
Coumaric acid	[M + H]^+^	6.26	C_9_H_8_O_3_	165.0546	165.0542	−2.8	147.0445, 77.0439
*p*-Hydroxybenzoic acid	[M − H]^–^	7.32	C_7_H_6_O_3_	137.0244	137.0247	2.0	93.0356, 65.0422
Protocatechuic acid	[M − H]^–^	7.42	C_7_H_6_O_4_	153.0193	153.0198	3.1	109.0292
*p*-Coumaric acid	[M − H]^–^	9.08	C_9_H_8_O_3_	163.0401	163.0403	1.2	119.0508, 93.0359
Ferulic acid	[M − H]^–^	9.75	C_10_H_10_O_4_	193.0506	193.0514	3.9	178.0264134.0379
**Flavonoids and derivatives**							
Gallocatechin	[M + H]^+^	5.67	C_15_H_14_O_7_	307.0812	307.0810	−1.0	163.0379, 139.0397
	[M − H]^–^	5.67	C_15_H_14_O_7_	305.0667	305.0664	−0.9	179.0352, 125.0247
Epigallocatechin	[M − H]^–^	6.73	C_15_H_14_O_7_	305.0667	305.0667	0.1	179.0344, 125.0248, 109.0301
	[M + H]^+^	6.74	C_15_H_14_O_7_	307.0812	307.0812	0.0	139.0392
Quercetin-3-*O*-glucoside	[M + H]^+^	6.89	C_21_H_20_O_12_	465.1028	465.1024	−0.8	303.0501
Procyanidin B2	[M − H]^–^	6.94	C_30_H_26_O_12_	577.1352	577.1347	−0.9	425.0873, 407.1035, 289.0719, 287.0552, 161.0243, 125.0249
	[M + H]^+^	6.95	C_30_H_26_O_12_	579.1497	579.1491	−1.1	561.1401, 427.1035, 291.0845, 139.0395
Quercetin-3-*O*-rutinoside (rutin)	[M + H]^+^	7.32	C_27_H_30_O_16_	611.1607	611.1581	−4.1	465.1019, 303.0468
Catechin	[M + H]^+^	7.40	C_15_H_14_O_6_	291.0863	291.0859	−1.5	139.0400, 123.0459
Quercetin-3-β-sophoroside	[M + H]^+^	7.77	C_27_H_30_O_17_	627.1556	627.1543	−2.0	465.1007, 303.0496
Epicatechin	[M − H]^–^	7.93	C_15_H_14_O_6_	289.0718	289.0720	0.8	245.0825, 203.0712, 161.0610, 151.0405, 125.0254, 109.0308
	[M + H]^+^	7.95	C_15_H_14_O_6_	291.0863	291.0863	−0.2	139.0400
Kaempferol-3-*O*-rutinoside	[M − H]^–^	8.34	C_27_H_30_O_15_	593.1512	593.1505	−1.1	447.0932, 285.0405
Quercetin-7-rhamnoside	[M + H]^+^	8.42	C_21_H_20_O_11_	449.1078	449.108	0.4	303.0521, 257.0513
Isorhamnetin-3-*O*-neohespeidoside	[M + H]^+^	8.44	C_28_H_32_O_16_	625.1763	625.1756	−1.1	317.0661
Kaempferol-3,7-*L*-dirhamnoside (Kaempferitrin)	[M + H]^+^	8.86	C_27_H_30_O_14_	579.1709	579.1710	0.3	433.1140, 287.0531
Myricetin-3-*O*-rhamnoside (Myricitrin)	[M − H]^–^	8.95	C_21_H_20_O_12_	463.0882	463.0870	−2.7	317.0275, 271.0240
Cyanidin-3-*O*-glucoside	[M + H]^+^	9.60	C_21_H_20_O_11_	449.1078	449.1080	0.3	287.0534
Quercetin-3-*O*-rhamnoside (Quercitrin)	[M + H]^+^	10.32	C_21_H_20_O_11_	449.1078	449.1083	1.0	303.0508
Myricetin	[M − H]^–^	10.45	C_15_H_10_O_8_	317.0303	317.0297	−1.8	151.0023
Quercetin	[M − H]^–^	11.80	C_15_H_10_O_7_	301.0354	301.0354	0.0	178.9977, 151.0044, 121.0301

**Table 3:** Quantitative results for major phenolic components in the extracts of 29 faba bean varieties by HPLC analysis.

Faba Bean Varieties	L-DOPA (mg/g DW)	Gallic Acid (mg/100 g DW)	Protocatechuic Acid (mg/100 g DW)	Gallocatechin (mg/100 g DW)	Epigallocatechin (mg/100 g DW)	Catechin (mg/100 g DW)	Procyanidin B2 (mg/100 g DW)	Rutin (mg/100 g DW)	Kaempferol-3-*O*-Rutinoside (mg/100 g DW)
Baihuahudou	14.18 ± 0.19	2.62 ± 0.03	8.63 ± 0.45	1.03 ± 0.01	20.98 ± 0.69	2.30 ± 0.42	1.43 ± 0.01	1.96 ± 0.02	1.57 ± 0.01
Chenghu 21	16.01 ± 0.57	2.92 ± 0.01	13.28 ± 0.13	2.05 ± 0.03	17.60 ± 0.02	3.15 ± 0.02	0.31 ± 0.02	4.59 ± 0.01	0.43 ± 0.01
Chenghudabai	16.86 ± 0.46	2.87 ± 0.04	15.15 ± 0.14	2.35 ± 0.43	18.13 ± 0.26	2.95 ± 0.07	0.27 ± 0.00	2.32 ± 0.01	0.37 ± 0.01
Chuzao 1	13.39 ± 0.31	2.53 ± 0.01	10.31 ± 0.05	1.43 ± 0.02	13.35 ± 0.31	1.90 ± 0.62	0.30 ± 0.02	8.23 ± 0.00	0.39 ± 0.01
Cican 1	13.90 ± 0.35	3.48 ± 0.08	8.80 ± 0.27	0.79 ± 1.36	20.18 ± 0.46	3.89 ± 0.12	1.28 ± 0.01	2.90 ± 0.21	0.64 ± 0.01
Dingxuancan 3	17.08 ± 0.20	3.15 ± 0.08	12.18 ± 0.38	2.74 ± 0.08	14.41 ± 0.01	1.97 ± 0.29	3.96 ± 0.01	5.56 ± 0.02	1.11 ± 0.03
Edou 1103	15.68 ± 0.25	3.50 ± 0.02	13.92 ± 0.14	1.45 ± 0.16	18.76 ± 0.36	4.36 ± 0.50	0.26 ± 0.02	1.91 ± 0.01	0.69 ± 0.01
Edou 1208	15.19 ± 0.34	3.25 ± 0.01	13.95 ± 0.09	2.08 ± 0.07	23.57 ± 0.01	3.94 ± 0.13	0.30 ± 0.00	2.78 ± 0.01	0.48 ± 0.01
Fengdou 11	12.99 ± 0.27	2.96 ± 0.01	10.21 ± 0.06	1.52 ± 0.28	14.88 ± 0.16	1.61 ± 0.24	0.24 ± 0.00	4.59 ± 0.10	0.31 ± 0.02
Fengdou 28	14.10 ± 0.43	2.87 ± 0.01	11.33 ± 0.05	3.86 ± 0.53	16.96 ± 0.29	2.32 ± 0.45	0.31 ± 0.00	5.14 ± 0.01	0.51 ± 0.03
Jianlixiaocandou	16.70 ± 0.71	2.11 ± 0.01	14.70 ± 0.16	1.56 ± 0.02	20.96 ± 0.03	2.64 ± 0.11	1.59 ± 0.01	2.67 ± 0.01	1.68 ± 0.01
Jingdou 5	12.88 ± 0.13	3.31 ± 0.08	7.72 ± 0.31	1.09 ± 0.01	16.08 ± 0.07	2.28 ± 0.01	5.47 ± 0.01	8.31 ± 0.01	0.27 ± 0.02
Jizhangcan 5	16.07 ± 0.27	3.75 ± 0.08	11.14 ± 0.24	4.94 ± 0.03	14.46 ± 0.07	3.55 ± 0.11	2.84 ± 0.30	3.90 ± 0.10	0.81 ± 0.02
Lican 3	13.07 ± 0.19	3.15 ± 0.06	8.04 ± 0.54	0.83 ± 0.70	13.38 ± 1.33	3.33 ± 0.49	1.36 ± 0.01	2.90 ± 0.05	0.38 ± 0.01
Lican 7	14.54 ± 0.13	3.86 ± 0.05	9.48 ± 0.55	4.01 ± 0.02	17.53 ± 0.04	6.08 ± 0.04	0.93 ± 0.00	1.57 ± 0.01	0.46 ± 0.00
Lincan 13	15.28 ± 0.18	2.89 ± 0.11	15.06 ± 0.25	1.23 ± 0.03	14.08 ± 0.13	2.12 ± 0.23	3.61 ± 0.01	5.05 ± 0.02	1.52 ± 0.02
Lincan15	14.65 ± 0.21	2.98 ± 0.15	14.54 ± 0.15	2.32 ± 0.02	11.55 ± 0.10	2.32 ± 0.09	1.67 ± 0.02	3.20 ± 0.02	0.79 ± 0.01
Qidou 2	16.86 ± 0.20	3.15 ± 0.07	12.11 ± 0.30	1.64 ± 0.02	19.73 ± 0.01	3.64 ± 0.03	1.34 ± 0.01	2.06 ± 0.01	2.16 ± 0.01
Qingcan 25	14.06 ± 0.09	2.94 ± 0.15	12.35 ± 0.12	2.38 ± 0.02	17.68 ± 0.08	1.99 ± 0.40	2.12 ± 0.01	5.91 ± 0.02	1.84 ± 0.02
Tongcanxian 20	12.49 ± 0.29	2.90 ± 0.09	11.33 ± 0.17	1.03 ± 0.06	17.04 ± 0.08	3.74 ± 0.07	1.21 ± 0.01	2.71 ± 0.01	0.35 ± 0.01
Tongcanxian 21	11.96 ± 0.08	2.80 ± 0.07	7.21 ± 0.37	0.00 ± 0.00	14.53 ± 0.02	5.10 ± 0.02	1.22 ± 0.00	2.06 ± 0.00	0.25 ± 0.01
Wancan 1	14.18 ± 0.02	2.87 ± 0.06	12.13 ± 0.28	3.09 ± 0.01	18.38 ± 0.03	3.17 ± 0.01	2.65 ± 0.01	4.19 ± 0.01	0.45 ± 0.01
Yican 4	14.75 ± 0.35	4.60 ± 0.02	12.12 ± 0.11	2.10 ± 0.34	18.11 ± 0.03	2.20 ± 0.00	0.20 ± 0.01	4.86 ± 0.01	0.45 ± 0.01
Yican 6	15.57 ± 0.44	3.74 ± 0.01	12.35 ± 0.10	3.91 ± 0.03	16.40 ± 0.07	2.79 ± 0.02	0.20 ± 0.03	7.92 ± 0.02	0.38 ± 0.01
Yucan 1	16.42 ± 0.31	3.45 ± 0.01	12.11 ± 0.48	2.54 ± 0.01	19.09 ± 0.01	3.58 ± 0.04	2.05 ± 0.01	2.86 ± 0.41	1.30 ± 0.02
Zhecan 1	14.54 ± 0.25	3.95 ± 0.04	12.43 ± 0.12	0.01 ± 0.00	19.84 ± 0.13	4.37 ± 0.02	1.09 ± 0.01	1.67 ± 0.02	0.37 ± 0.01
Zhongjiang changxiu	17.93 ± 0.21	3.60 ± 0.05	13.14 ± 0.42	3.52 ± 0.04	14.56 ± 1.44	3.41 ± 0.57	1.80 ± 0.01	2.36 ± 0.00	0.17 ± 0.00
2016-831	16.47 ± 0.17	3.21 ± 0.07	16.37 ± 0.19	1.05 ± 0.02	18.68 ± 0.07	2.43 ± 0.05	1.01 ± 0.01	2.23 ± 0.02	1.68 ± 0.05
P16-06-3	12.49 ± 0.37	3.18 ± 0.07	11.28 ± 0.07	1.45 ± 0.38	17.10 ± 0.24	3.66 ± 0.14	1.20 ± 0.06	2.84 ± 0.02	0.76 ± 0.02

**Table 4:** Antioxidant capacity and enzyme inhibitory effects of L-DOPA.

Sample	DPPH (µmol TE/g)	ABTS (µmol TE/g)	FRAP (mmol FE/g)	α-Amylase IC_50_ (μmol/L)	α-Glucosidase IC_50_ (μmol/L)
L-DOPA	3.66 ± 0.39	3.21 ± 0.33	9.75 ± 0.01	24.87 ± 2.21	16.66 ± 0.69
Positive control	3050.00 ± 83.89	4271.11 ± 3.85	13.50 ± 0.33	0.54 ± 0.08	1.24 ± 0.15

Ascorbic acid (vitamin C) was used as the positive control for DPPH, ABTS, and FRAP, while acarbose was used as the positive control for α-amylase and α-glucosidase inhibitory effects. DPPH, DPPH radical scavenging activity; ABTS, ABTS radical cation scavenging activity; FRAP, ferric reducing antioxidant power; TE, Trolox equivalent; FE, Fe^2+^ equivalent; IC_50_, half (50%) maximal inhibitory concentration.

## Data Availability

The data presented in this study are available upon request.
